# Stems and leaves of angiosperms follow a convex trade‐off to optimise hydraulic safety and efficiency

**DOI:** 10.1111/nph.70895

**Published:** 2026-01-11

**Authors:** Swetlana Kreinert, Luciano Pereira, Lucian Kaack, Marcela T. Miranda, Rafael V. Ribeiro, Steven Jansen

**Affiliations:** ^1^ Institute of Botany Ulm University Albert‐Einstein‐Allee 11 89081 Ulm Germany; ^2^ Laboratory of Crop Physiology, Department of Plant Biology, Institute of Biology State University of Campinas (UNICAMP) PO Box 6109 13083‐970 Campinas São Paulo Brazil

**Keywords:** embolism, hydraulic conductivity, pit membrane, safety and efficiency, xylem

## Disclaimer

The New Phytologist Foundation remains neutral with regard to jurisdictional claims in maps and in any institutional affiliations.

## Comment on Jin *et al.* (2024) 'Precipitation, solar radiation, and their interaction modify leaf hydraulic efficiency‐safety trade‐off across angiosperms at the global scale'

A long‐standing question in studies on plant water relations addresses the relationship between transport efficiency and safety. Can xylem conduits in a plant species be hydraulically highly efficient, conducting a large amount of water per unit of leaf area, and simultaneously be safe to avoid embolism formation via interconnected neighbouring conduits? The answer to this question requires an insightful, complex understanding of various processes and is not immediately apparent.

Hydraulic safety of conduits relates to their capacity to suppress disruption of water transport by large gas bubbles, which is known to be the largely irreversible process of embolism formation. High levels of embolism may contribute to tissue desiccation and plant mortality (Adams *et al*., 2017). Embolism resistance and the estimated water potential corresponding to either 50% loss of hydraulic conductivity or 50% of the maximum amount of embolised conduits, is a direct and mechanistic parameter of hydraulic safety. There is also clear evidence that the likelihood of embolism propagation increases with lowering water potential in the xylem, especially under drought. Yet, the ability to deliver plenty of water to leaves while maintaining a low water potential gradient between roots and transpiring leaves allows plants to keep their stomata open over long periods, making gas exchange for photosynthesis less affected by daily changes in vapour pressure deficit (VPD) and/or soil water deficit.

Hydraulic efficiency, which is estimated as hydraulic conductivity and therefore the amount of water transported per unit pressure gradient, is directly related to gas exchange, but forms a much more complex trait (Brodribb *et al*., 2007; Pereira *et al*., 2023). In leaves, for example, the nature of the mesophyll pathway and its length strongly affect the hydraulic conductivity, making the extravascular resistance the unifying limit to hydraulic efficiency (Brodribb *et al*., 2007). In secondary xylem, the hydraulic conductivity is determined by various xylem anatomical parameters. The flow is directly related to the total intervessel pit surface area (*A*_p_) and inversely related to intervessel pit membrane thickness (*T*_pm_). Besides *A*_p_ and *T*_pm_, vessel diameter and vessel length are also strongly related to efficient water transport in plants (Franklin *et al*., 2023; Pereira *et al*., 2023; Kreinert *et al*., 2025). These parameters, particularly *T*_pm_ (Kaack *et al*., 2021), can also determine hydraulic safety, which means that optimisation of water transport can be directly linked to embolism resistance (Hacke & Sperry, 2001; Franklin *et al*., 2023). Therefore, a trade‐off would be expected as plants cannot be both highly efficient and safe (Gleason *et al*., 2016; Pereira *et al*., 2023). While the occurrence of fibriform vessels (i.e. fibre‐like, short and narrow vessels) could increase hydraulic efficiency, while increasing safety (Vilagrosa *et al*., 2014), it is not well known how narrow vessels or tracheids affect intervessel connectivity and therefore hydraulic efficiency, since vessel lumen fractions are relatively constant (Zanne *et al*., 2010; Morris *et al*., 2016).

Since safety and efficiency of water transport are tightly linked to leaf water status and stomatal behaviour (Scoffoni *et al*., 2016), eco‐evolutionary optimality (EEO) principles may have invoked both aspects of long‐distance water transport in plants, while an uncompetitive trait combination would be eliminated by natural selection (Franklin *et al*., 2023). Therefore, hydraulic traits have likely adapted towards the maximisation of plant fitness in their environment, leading to a hydraulic efficiency‐safety trade‐off.

The idea behind a safety‐efficiency trade‐off as part of a wood economics spectrum is appealing and intuitive (Chave *et al*., 2009), but the available evidence is less clear. In fact, at a global scale, the safety‐efficiency estimated on stems was classified as a weak trade‐off (Gleason *et al*., 2016), and a similar conclusion was recently reached for leaves (Jin *et al*., 2024). Jin *et al*. showed no clear linear relationship between embolism resistance and leaf conductance. While we recognise the great effort in data compilation and the opportunities these bring for analyses on a global scale, we emphasise in this commentary that the boundary nature of the hydraulic efficiency‐safety trade‐off is nonlinear and that both traits can be uncoupled due to the function of bordered pit characteristics. Our novel insights into the structural functional aspects of bordered pits are needed to better understand the convex boundary of this relationship, with extreme safety and efficiency strategies being favoured (Ehrlich *et al*., 2017) without the need for a direct linear correlation. The reason why hydraulic safety and efficiency can be uncoupled is that both properties are tightly related to the characteristics of xylem end walls, which interconnect neighbouring vessels via pit membranes in bordered pits. Although the functional roles of interconduit pits and pit membranes have been suggested previously (Tyree & Sperry, 1989; Tyree & Zimmermann, 2002; Choat *et al*., 2008), the essential role of their pit membranes as mesoporous media has only recently been demonstrated (Kaack *et al*., 2021; Pereira *et al*., 2023).

## The effects of vessel dimensions on hydraulic safety and efficiency

Hydraulic conductivity relates to the fourth power of the vessel diameter according to the Hagen‐Poiseuille equation (Sperry *et al*., 2005, 2006). Since vessels have a finite length and are known to be interconnected, especially at their starting and ending tails (Wason *et al*., 2017; Wason *et al*., 2021; Bouda *et al*., 2022), the end wall resistance needs to be considered when estimating hydraulic conductivity. Similarly, the vessel length distribution should be considered for correct estimations of lumen conductivity and specific conductivity (Kreinert *et al*., 2025). Experimental data on the specific hydraulic conductivity (*K*_s_) of a particular stem segment, as compiled by Gleason *et al*. (2016), do not consider vessel length distributions, which have been challenging to measure based on traditional methods. Normalisation of conductivities to segment length is a frequent approach (Choat *et al*., 2008) but is known to be prone to artefacts or inconsistencies (Kolb & Sperry, 1999), while normalisation per unit leaf area could also provide another approach to normalise conductivity data (Olson *et al*., 2021). In addition, *K*_s_ relates to other functions of the wood space use since *K*_s_ can be related to the sapwood or stem area (Bittencourt *et al*., 2016). Space use for storing water is also important for plant water relations and contributes to a desiccation delay strategy (Tyree *et al*., 2003).

Xylem sap must also cross bordered pits in end walls, which represent *c*. 50% of the total hydraulic resistance (Sperry *et al*., 2005; Hacke *et al*., 2006; Choat *et al*., 2008). Two useful parameters that quantify this resistance include: (1) the total intervessel pit membrane area (*A*_p_) for a given vessel, which reflects the intervessel conductivity; and (2) the intervessel pit membrane thickness (*T*_pm_) (Pereira *et al*., 2023; Zhang *et al*., 2024). While thick pit membranes are mechanistically related to high embolism resistance in interspecific analyses (Kaack *et al*., 2021; Miranda *et al*., 2024), a large intervessel pit membrane area and thin pit membranes are related to low hydraulic resistivity and, consequently, to high hydraulic efficiency (Pereira *et al*., 2023). Yet, embolism resistance may not be affected by the total pit membrane area of a vessel (Kaack *et al*., 2021), and even thin pit membranes could provide sufficient safety against embolism formation under various environmental conditions. Thus, any safety‐efficiency trade‐off of water transport through vessels should consider end wall characteristics such as *T*_pm_ and *A*_p_.

Applying Darcy's law to estimate flow through a vessel end wall revealed a convex relationship driven by intervessel pits, where efficiency was quantified based on conductivity, and safety based on the pressure difference (∆*P*) across a pit membrane (Pereira *et al*., 2023). A consequence of this nonlinear trade‐off is the potential lack of a direct coupling between safety and efficiency. Small changes in *A*_p_ or *T*_pm_ could substantially change the hydraulic safety or efficiency and may allow plants to adapt their water transport strategies to varying environmental conditions (Pereira *et al*., 2023). Indeed, increased grouping of vessels is likely associated with an increase in *A*_p_ and has repeatedly been observed in closely related species that grow along a gradient with increasing drought exposure and have a non‐conductive ground tissue (Carlquist, 1966, 1984; Johnson *et al*., 2023). If pit membranes are relatively thick, then the effect of *A*_p_ on safety is limited, and *A*_p_ would largely affect efficiency instead of safety. However, the effect of *A*_p_ on safety can be large when pit membranes are thin, but then *A*_p_ may not affect efficiency equally. When values of *A*_p_ and *T*_pm_ are within a range of average values, changes in *A*_p_ or *T*_pm_ likely need to be substantial to significantly affect efficiency or safety (Pereira *et al*., 2023).

## Hydraulic constraints in xylem and outside‐xylem

The proxy for efficiency used by Jin *et al*. (2024) is the leaf conductivity (*K*_leaf_), which was estimated using the evaporative flux method (Sack *et al*., 2002). Thus, it also included other resistivities outside the xylem (extraxylary resistivity), as discussed by the authors. The extraxylary resistivity may be related to a desiccation delay strategy since it can prevent water loss. Specifically, traits such as leaf mass per area, which relate to extraxylary path length and vein density, were found to be more strongly related to hydraulic safety, without proportionally sacrificing efficiency, leading to a weak trade‐off between *K*_leaf_ and *P*_50_, and therefore no strong safety‐efficiency trade‐off (Jin *et al*., 2024). Although we agree with the authors that extraxylary resistivity, including resistivity by aquaporins, plasmodesmata, apoplastic barriers, and stomata, can be related to the rate of leaf desiccation, the extraxylary conductivity is a direct continuation of xylem hydraulic conductivity within the entire water pathway from the plant to the atmosphere, including the leaf boundary layer (Maurel *et al*., 2015; Tee & Faulkner, 2024). Within the soil–plant continuum, we suggest that water transport through xylem and outside‐xylem tissue (including both leaves and roots) can be understood based on two theoretical, dynamic perspectives. Plants may exhibit a strong hydraulic limitation at the extraxylary level during drought, when its very low conductivity also limits transport through xylem vessels. During periods of transpiration, however, hydraulic properties of xylem and outside‐xylem likely scale in a way to optimise whole‐plant hydraulic resistivity and efficiency within a soil–plant–atmosphere continuum.

If the xylem pathway and/or the outside‐xylem pathway would provide any major hydraulic limitation, this would imply that plants are unavoidably overbuilding their pathway, while wasting carbon investment to conduct water with a higher resistance to the leaf mesophyll cells than what would be needed to keep these cells sufficiently hydrated (hypothesis 1). On the other hand, a lack of any hydraulic limitation would suggest that a species only invests as much carbon as is required to guarantee a proportional conductivity throughout the plant body (hypothesis 2). Based on the concept of eco‐evolutionary optimality, we have reasons to believe that there is no hydraulic limitation within the transport pathway of a plant, and that the second, whole‐plant hydraulic scaling hypothesis is likely. Considering the conduit widening concept from tip‐to‐base, a proportional scaling between vessel lumen resistance and end wall resistance has already been suggested (Olson *et al*., 2014, 2021; Anfodillo & Olson, 2024). Dynamic changes in the outside‐xylem pathway, however, rely on living tissue, which may control hydraulic resistance by opening or closing various valve‐like systems, including stomata. No xylem hydraulic limitation, but a tip‐to‐base scaling was recently described for a *Fagus sylvatica* sapling (Kreinert *et al*., 2025), although leaves were not included in this study.

We tested the above two hypotheses based on the database of Jin *et al*. (2024) for leaf‐specific conductivity (*K*_leaf_), combined with stem anatomical data from Kaack *et al*. (2021). This approach enabled us to test if the lack of a xylem hydraulic limitation for stems also applies to leaves. If the xylem pathway would not be limiting hydraulic conductivity, *K*_leaf_ should be proportional to vessel lumen (*K*_h_) and end wall (*K*_w_) conductivities across species. At the same time, embolism resistance (*P*_50_) should be related to *K*_w_, since *P*_50_ is strongly affected by *T*_pm_, a trait directly related to embolism resistance (Kaack *et al*., 2021; Miranda *et al*., 2024).

## Testing the convex safety‐efficiency trade‐off

To assess whether the convex relationship between hydraulic safety and efficiency previously observed for stems (Pereira *et al*., 2023) also extends to leaves, we examined whether leaf hydraulic conductance (*K*_leaf_) could also be convexly related to hydraulic safety (*P*_50_). Such a relationship would indicate a hydraulic scaling between stem and leaf conductivity, suggesting optimisation of water transport across the xylem pathway within the soil–plant‐atmosphere continuum. We modelled the relationship between safety (response variable y; either *P*_50_ or the pressure difference across end walls; ∆*P*) and efficiency (predictor *x*: either *K*_leaf_ or end wall conductivity; *K*_w_) using a nonlinear asymptotic function (Eqn 1):

(Eqn 1) $$y=a\times \left(1-e^{-\mathrm{bx}}\right)$$

where *a* was the asymptotic maximum value of *y*, and *b* was a rate parameter responsible for the velocity at which *y* approached *a*. Before analysis, all variables were normalised to their minimum–maximum values, resulting in relative values between 0 and 1 to ensure comparability across the traits.

For reference, we also fitted linear models of the form:

(Eqn 2) y=mx+b

where *m* is the slope and *b* is the intercept.

**Fig. 1:**
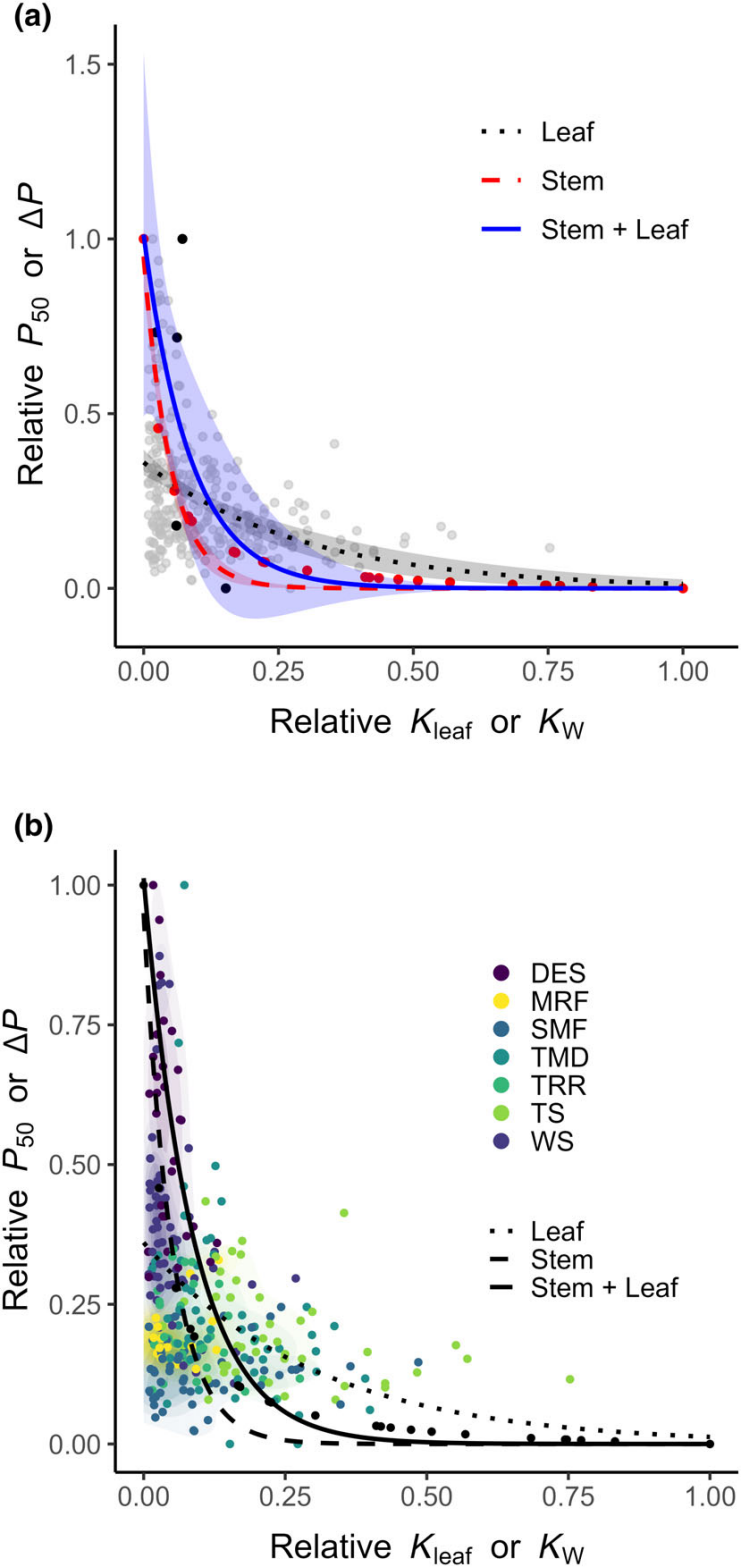
Relationships between stem and leaf hydraulic traits across species, derived from independent and overlapping datasets as well as their biome‐specific variation. (a) The grey points represent the leaf dataset (*n* = 280; Jin *et al*., 2024), the red points represent the stem database (*n* = 22; Kaack *et al*., 2021), and the black dots represent the species presented in both datasets (*n* = 5). The black fit is based on the leaf database (Jin *et al*., 2024), the red fit is based on the stem database (Kaack *et al*., 2021), and the blue fit represents the joint species from both databases. All fits include a 95% confidence interval. (b) Relationship between relative values of *K*_leaf_ vs *P*_50_ and *K*_w_ vs end wall pressure difference (Δ*P*). A nonlinear, convex fit shows an improved explanation for the observed triangular relationship between *x* and *y*. The points were coloured by biomes: desert (DES), mountain rainforest (MRF), submontane rainforest (SMF), tropical montane rainforest (TMD), tropical rainforest (TRR), tundra shrubland (TS), and wetland shrubland (WS).

Separate nonlinear (nls) (Fig. 1) and linear (lm) models were fitted in three groups: the leaf database (Leaf, L) (Jin *et al*., 2024), the xylem (Stem, S) dataset (Kaack *et al*., 2021), and a combined dataset composed of species included in the leaf and stem datasets (Stem + Leaf, S + L). For the combined dataset, we used paired measurements of *P*_50_/∆*P* and *K*_leaf_/*K*_w_ for the same species, yielding a standardised dataset with both safety and efficiency traits (*n* = 5, representing five measurements per each tissue (leaf and stem) examined).

Analysis of the data presented by biomes reveals a separation across the safety and efficiency trade‐off suggesting specific hydraulic strategies of the plants (Fig. 1a). Species found in the desert accumulate on the safe side of the trade‐off with either high ∆*P* or low *P*_50_ values, while species of the submontane, montane, or tropical rainforest are more spread across the trade‐off, following more the efficient axis as well as reduced ∆*P* and more positive (higher) *P*_50_ values.

The model parameters were estimated by nonlinear least squares analyses, shown together with SE, and significance levels in Table 1. The nls fits showed significant parameter estimates for both leaf and stem datasets (*P* < 0.05), with low residual SE and AIC values, indicating good model performance and limited overfitting. The combined dataset exhibited marginal significance for parameter *b* (*P* = 0.0405), likely due to the small sample size relative to the leaf or xylem datasets.

**Table 1:** Summary of model outcomes.

	Parameter	Estimate	SE	*t* value	*P* value	Residual_SE	*R* ^2^	AIC	df
nls Leaf	*a*	0.3605	0.0187	19.2895	0	0.1615	0.155	−222.31	278
nls Leaf	*b*	3.3498	0.5673	5.9045	1.03E‐08	0.1615	0.155	−222.31	278
nls Stem	*a*	0.9499	0.0419	22.6839	8.88E‐16	0.0444	0.96	−70.72	20
nls Stem	*b*	19.8555	1.5952	12.4467	7.11E‐11	0.0444	0.96	−70.72	20
nls Stem + Leaf	*a*	1.0125	0.2256	4.48969	0.00203	0.2606	0.64	5.25	8
nls Stem + Leaf	*b*	11.5050	4.7136	2.4408	0.040514	0.2606	0.64	5.25	8
lm Leaf	Intercept	0.3253	0.014	23.2604	3.48E‐67	0.1641	0.128	−213.48	278
lm Leaf	rel_*K*_leaf_	−0.5578	0.0875	−6.3755	7.56E‐10	0.1641	0.128	−213.48	278
lm Stem	Intercept	0.3153	0.0667	4.728734	0.000129	0.1815	0.382	−8.74	20
lm Stem	rel_*K*_w_	−0.4695	0.1337	−3.51278	0.002189	0.1815	0.382	−8.74	20
lm Stem + Leaf	Intercept	0.5651	0.1369	4.126811	0.003313	0.3558	0.326	11.48	8
lm Stem + Leaf	*x*	−0.7268	0.3694	−1.96766	0.08465	0.3558	0.326	11.48	8

Nonlinear models (nls) estimated parameters *a* and *b*, and a linear model (lm) estimated intercepts and slopes. For each model, the Akaike information criterion (AIC) and degrees of freedom (df) were reported. Statistically significant predictors (*P* < 0.05) were observed in most models with only one exception, that is the linear model for Stem + Leaf dataset. The stem and leaf datasets were based on Jin *et al*. (2024) and Kaack *et al*. (2021), respectively. *K*_w_, end wall conductivity of xylem vessels; *K*_leaf_, leaf specific conductivity (including xylem and outside‐xylem tissue).

**Fig. 2:**
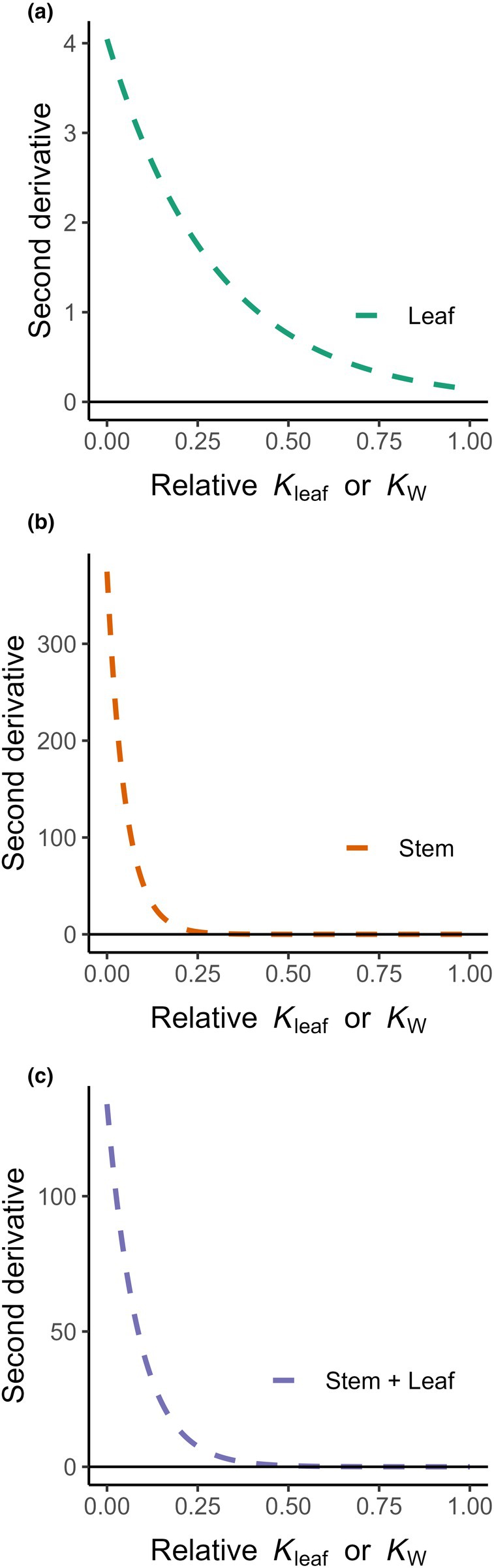
Second derivative for each subset of data – leaf (a, *n* = 280), stem (b, *n* = 22), and the leaf + stem species (c, *n* = 5). *F*″ (*x*) ≥ 0 for all *x* in the given interval, confirming that the data were convex. A non‐decreasing slope (negative values) means that the function curve went ‘upward’, which matches the geometric intuition behind convexity. The stem and leaf datasets were based on Jin *et al*. (2024) and Kaack *et al*. (2021), respectively.

The convexity of the nls fits was supported by positive second derivatives across all datasets (Fig. 2), confirming that each function curved upward, consistent with the geometric definition of convexity that *F*″(*x*) was ≥ 0 for all *x* in the interval of interest. Calculation of the second derivatives is used to assess the convexity of a function by measuring the rate of slope change. A positive second derivative indicated that the function was curving upwards, which was the defining characteristic of a convex function. In Fig. 2, all three datasets displayed second derivative values above zero across the range of relative *K*_leaf_ or *K*_w_. The strictly positive values across all datasets, despite differences in sample size, provided mathematical evidence that each model fulfilled the condition for convexity. In other words, the triangle‐like distribution of data points observed could be captured more accurately by nonlinear models than by linear ones. This convexity explained the tapering pattern in datasets, which linear models failed to adequately represent, especially in the presence of low relative *K*_leaf_ or *K*_w_ values.

The difference in the steepness and shape of the presented second derivative curves arises from differences in the parameters *a* (scaling factor) and *b* (rate constant) that are estimated for each model. A larger *b* value produces a steeper and more rapidly decaying curve, indicating that the underlying model with the nonlinear function (Fig. 1) decreases faster with increasing relative conductivity. These differences in steepness are reflected by the intrinsic model parameters that describe how efficiently each tissue conducts water within the safety and efficiency spectrum.

Biologically, the parameter *b* captures how sensitive water transport efficiency and, therefore, relative *K*_leaf_ or *K*_w_ is. In leaves, smaller *b* values represent gradual declines, meaning that leaf hydraulic conductivity decreases more slowly compared to the xylem tissue as water deficit increases. In addition, the difference in the steepness could be attributed to the method used to obtain the respective conductivities. While for the end wall conductivity we use xylem anatomical parameters, the evaporative flux method was used for leaf conductivities (Jin *et al*., 2024). The combination of the datasets (Figs 1, 2c) resulted in a *b* value being in‐between the two separate datasets and was a result of the combination of conductivities in parallel, meaning that the total conductivity was the sum of the conductivities in each tissue or segment. Hence, we found a convex relationship between species for stem, leaf, and the combination of leaf and stem.

Lastly, we compared the goodness of fit for the models using the Akaike information criterion (ΔAIC = AIC (lm) − AIC (nls)). ΔAIC values above 10 provided strong evidence against the linear models for the stem dataset. ΔAIC values between 2 and 10 indicate considerably better support for the model with a lower AIC (Table 1). Specifically, the comparison revealed ΔAIC values of 8.82 for the leaf dataset, 61.98 for the stem dataset, and 6.23 for the combined dataset. The positive ΔAIC values across all the datasets indicate that the nls models provided a better fit than the lm model, suggesting that the nls model explains the data distribution better than the lm model.

The convex relationship between *K*_leaf_ and *P*_50_ suggests that the same trade‐off observed in stems, which is due to functional implications of pit membrane thickness (*T*_pm_) and pit area (*A*_p_) at the vessel end wall level, also occurs at the leaf level. This functional analogy between stems and leaves is not novel and has previously been suggested based on similarities in the comparative anatomy of stems and leaves (Dickison, 2000). A whole‐plant hydraulic scaling hypothesis seems plausible given the functional nature of the soil–plant–atmosphere continuum, in which, for instance, a hydraulically efficient stem should be complemented by an efficient leaf, including a high extraxylary conductivity. Conversely, hydraulically safe stem xylem cannot be highly efficient due to relatively high *T*_pm_ and low *A*_p_ values, and a relatively low conductivity is likely to occur at the leaf vessel level and outside‐xylem level. Although this hypothesis still requires direct evidence, there is clear support for proportional conductivities (including *K*_h_ and *K*_w_) from the stem base to the terminal branches (Kreinert *et al*., 2025), and the results presented here suggest that this proportionality may extend to the leaf level, including the outside‐xylem tissue. Future work would also be needed to test how outside‐xylem tissue in roots follows a whole‐plant hydraulic scaling pattern.

## Conclusion

Our findings indicate that a hydraulic safety‐efficiency trade‐off is not linear but convex, and applies to stem and leaf xylem and to non‐xylem tissue in leaves. The trade‐off in xylem safety and efficiency is largely determined by water flow across intervessel pit membranes, as proposed by Pereira *et al*. (2023). Furthermore, the hydraulic system of angiosperms exhibits an optimised and proportional coordination between xylem and extra‐xylary water transport, without apparent constraints from stems to leaves (Kreinert *et al*., 2025). The pit membrane traits *A*_p_ and *T*_pm_ emerge as a mechanistic link through which plants optimise their hydraulic performance of xylem in response to environmental conditions and resource limitations.

## Competing interests

None declared.

## Author contributions

SK and LP conceived the idea and drafted the first version. LK contributed to data analysis and interpretation. SJ, LK, RVR and MTM contributed to the manuscript writing. SK and LP contributed equally to this work.

## Supporting information


**Dataset S1** Raw dataset (Jin *et al*., 2024) supporting the results presented in the main text.

**Dataset S2** Raw dataset (Kaack *et al*., 2021) supporting the results presented in the main text.

**Dataset S3** Code used to perform the analysis presented in the main text.
Please note: Wiley is not responsible for the content or functionality of any Supporting Information supplied by the authors. Any queries (other than missing material) should be directed to the *New Phytologist* Central Office.

## Data Availability

The data that support the findings of this study are available in the Supporting Information of this article (Datasets S1– S3).
